# Gastrin-releasing peptide receptor: a promising new biomarker to identify cervical precursor lesions and cancer

**DOI:** 10.61622/rbgo/2025rbgo4

**Published:** 2025-03-17

**Authors:** Martina Lichtenfels, Rafaella Almeida Lima Nunes, Rossana Veronica Mendoza López, Camila Alves da Silva, Luiz Carlos Zeferino, Vanesca de Souza Lino, Adhemar Longatto-Filho, Louise De Brot, Silvia Helena Rabelo-Santos, Daniela Baumann Cornelio, Enrique Boccardo, Caroline Brunetto de Farias, Lara Termini

**Affiliations:** 1 Ziel Biosciences Porto Alegre Rio Grande do Sul Brazil Ziel Biosciences, Porto Alegre, Rio Grande do Sul, Brazil.; 2 Universidade de Sao Paulo Faculdade de Medicina Hospital das Clinicas HCFMUSP São Paulo Brazil Center for Translational Research in Oncology (LIM/24), Instituto do Cancer do Estado de Sao Paulo, Hospital das Clinicas HCFMUSP, Faculdade de Medicina, Universidade de Sao Paulo, São Paulo, Brazil.; 3 Universidade de Sao Paulo Comprehensive Center for Precision Oncology São Paulo Brazil Comprehensive Center for Precision Oncology (C2PO), Universidade de Sao Paulo, São Paulo, Brazil.; 4 State University of Campinas Faculty of Medical Sciences Division of Gynecologic and Breast Oncology São Paulo Brazil Department of Obstetrics and Gynecology, Division of Gynecologic and Breast Oncology, Faculty of Medical Sciences, State University of Campinas (UNICAMP – Universidade Estadual de Campinas), São Paulo, Brazil.; 5 Universidade de São Paulo Instituto de Ciências Biomédicas Department of Microbiology São Paulo Brazil Department of Microbiology, Instituto de Ciências Biomédicas, Universidade de São Paulo, São Paulo, Brazil.; 6 University of São Paulo School of Medicine Department of Pathology São Paulo Brazil Laboratory of Medical Investigation (LIM) 14, Department of Pathology, School of Medicine, University of São Paulo, São Paulo, Brazil;; 7 University of Minho School of Medicine Life and Health Sciences Research Institute Braga Portugal Life and Health Sciences Research Institute, School of Medicine, University of Minho, Braga, Portugal; ICVS/3B's;; 8 Pio XII Foundation Barretos Cancer Hospital Molecular Oncology Research Center São Paulo Brazil Molecular Oncology Research Center, Barretos Cancer Hospital, Pio XII Foundation, Barretos, São Paulo, Brazil.; 9 AC Camargo Cancer Center Department of Pathology São Paulo Brazil Department of Pathology, AC Camargo Cancer Center, São Paulo, Brazil.; 10 Universidade Federal de Goiás Faculdade de Farmácia Goiás Brazil Faculdade de Farmácia, Universidade Federal de Goiás (UFG), Goiás, Brazil.; 11 Irmandade Santa Casa de Porto Alegre Rio Grande do Sul Brazil Irmandade Santa Casa de Porto Alegre, Rio Grande do Sul, Brazil

**Keywords:** Uterine cervical neoplasms, Gastrin-releasing peptide receptor, Human papillomavirus, Papillomavirus infections, Uterine cervical dysplasia, Oncogenes, Carcinoma, squamous cell, Adenocarcinoma

## Abstract

**Objective:**

This study aimed to verify the relation between gastrin-releasing peptide receptor (GRPR), oncogenic Human Papillomavirus (HPV) and cervical lesions severity.

**Methods:**

GRPR mRNA levels were evaluated in cervical cancer-derived cell lines and in primary keratinocytes expressing HPV16 oncogenes by RT-PCR. GRPR protein expression was assessed by immunohistochemistry in organotypic cell cultures derived from keratinocytes transduced with HPV16 oncogenes and in 208 cervical samples, including 59 non-neoplastic tissue, 28 cervical intraepithelial neoplasia grade 3 (CIN3), 44 squamous cell carcinomas (SCC) and 77 adenocarcinomas (ADC). Generic primers (GP5+/GP6+) were used to identify HPV infection in tissue samples. Experiments involving cell lines were analyzed through non-parametric tests (Kruskal Wallis), and Fisher's Exact Test for human tissues samples. All statistical tests were considered significant at p <0.05. Immunohistochemical evaluation was conducted independently and blindly by two observers (AD- LO). Any discordant findings were resolved through discussion to reach a consensus score.

**Results:**

GRPR mRNA levels were not increased in cells expressing HPV16 or HPV18 oncogenes. However, at the protein level, GRPR was upregulated in organotypic cell cultures containing HPV oncogenes. Besides, it was identified an association between GRPR expression and cervical lesion severity (p < 0.0001). The detection rate of high-risk HPV DNA was directly correlated with cervical disease. Nonetheless, HPV infection was not directly associated with GRPR in cervical samples.

**Conclusion:**

GRPR expression is highly predictive of cervical lesion severity, irrespective of HPV infection and might contribute to improving patient's therapeutic management as well as being used a marker of disease progression.

## Introduction

Cervical cancer is the fourth most common cancer in women worldwide and the second one when considering women aged between 15 to 44 years.^(1-3)^ Squamous cell carcinomas (SCC) and adenocarcinomas (ADC) account for 75-85% and 11-25% of all cases, respectively.^(3,4)^

Human Papillomavirus (HPV) infection is the primary etiological factor of cervical cancer, with approximately 70% of cases related to high-risk HPV16 and 18.^(3,5-7)^ Vaccination and screening strategies are critical tools for HPV-associated cancer prevention. However, developing countries have shown increased incidence and mortality rates due to limited structural and financial resources to support population screening programs, including those involving the Papanicolaou test (Pap test).^(8-10)^

Taking into account the low sensitivity of Pap test and difficulties in full cytology implementation in certain regions, HPV DNA detection emerges as a more sensitive and reproducible alternative, and a cost-effective option for screening. However, the specificity of molecular HPV tests depends on theirs application in a very well-defined population (over 25 or 30 years old depending on guidelines and target population) or together with other tests (HPV/Pap cotest, for example).^(11-13)^ Additionally, most HPV infections are eliminated by human immune system, and the presence of high-risk HPV (hrHPV) DNA represents an important risk factor, but not an ultimate marker for precancerous lesions or cancer. Therefore, additional test to detect precancerous lesions and cancer are still necessary.^(14)^

In this scenario, the identification and use of new biomarkers would increase both sensitivity and specificity of oncological cytology and HPV testing, enhancing the quality of screening programs, with an adequate clinical management of patients, especially in monitoring precancerous lesions.

Gastrin-releasing peptide (GRP) exerts numerous physiological functions including gastrointestinal hormone release, smooth muscle cell contraction and pancreatic enzyme secretion, in addition to acting as a central nervous system neurotransmitter. Its effects are mediated through the gastrin-releasing peptide receptor (GRPR), a member of the G-protein receptor superfamily.^(15,16)^ GRP and GRPR have been linked to growth-stimulatory effects in several types of cancer. Among GRP-associated factors involved in carcinogenesis, effects on morphogenesis, angiogenesis, cell migration, cell adhesion, cell proliferation and cell growth can be mentioned, suggesting a possible role in cancer development.^(17-20)^GRPR has been identified as a potentially useful biomarker for cervical cancer detection. High GRPR expression has been observed in cervical tumors, while its levels are usually low in healthy tissues.^(16,21,22)^ Previous studies have shown that GRPR immunostaining demonstrated high sensitivity, specificity, and accuracy in detecting cervical invasive cancer.^(21)^ However, there is a lack of data exploring the possible correlation between HPV infection and GRPR in cervical cancer.

The aim of this study was to verify the relation between GRPR expression, HPV infection and cervical lesion severity.

## Methods

Tissue samples were selected from pathology records obtained from women consecutively treated at the Women's Hospital, Campinas State University, Brazil, between 2005 and 2011. Cervical samples with a confirmed histological diagnosis from the institutions' pathology departments and sufficient formalin-fixed paraffin-embedded tissue for additional immunohistochemical assays were included. We investigated the protein levels of GRPR in 208 cervical samples including cervicitis, cervical intraepithelial neoplasia grade 3 (CIN3), SCC and ADC, all assessed using GRPR immunohistochemistry staining.

Cervical cancer-derived cell lines SiHa (HPV16, ATCC #HTB-35), HeLa (HPV18, ATCC #CCL-2) and C33 (HPV negative, ATCC #HTB-31) were cultured in Eagle's minimal essential medium (MEM) (Invitrogen, Carlsbad, CA, USA) supplemented with 10% bovine fetal serum (BFS) (Cultilab, Campinas, SP, Brazil) and maintained at 37°C and 5% CO_2_. Low-passage pooled neonatal foreskin keratinocytes (Lonza Walkersville, Inc., Walkersville, MD, USA) were grown in KGM-Gold™ (Keratinocyte Growth Medium, Lonza, Switzerland). At passage one, cells were acutely infected with recombinant pLXSN retroviruses either empty or containing HPV16 E6 and/or E7 oncogenes and expressing the neomycin selection marker. After 24 h, cells were selected with 300 µg/mL of G418 for 2 days, when 100% of mocked infected controls were dead. Surviving cells were amplified and used in monolayer experiments or to seed the epithelial raft cultures without extensive passage. Briefly, parental and transduced keratinocytes were seeded on top dermal equivalents (2 × 10^5^ cells/equivalent) composed of rat-tail type 1 collagen (Corning Inc., Corning, NY, USA) and 3T3-J2 fibroblasts. After 24 h, rafts were transferred to the medium–air interface and maintained for 9 days to allow cell growth and tissue stratification. Rafts were fixed in formaldehyde 2%, paraffin-embedded, and tissue sections were obtained for histological analysis or immunohistochemistry (IHC). Dr. Denise Galloway gently donated recombinant pLXSN-based retroviral vectors for expression of HPV16 oncogenes (Human Biology Division and Public Health Sciences at Fred Hutchinson Cancer Research Center in Seattle, WA, U.S.A.).

Total RNA was extracted from cervical cancer-derived lineages (C33, SiHa and HeLa) and from keratinocytes transduced or not with HPV16 oncogenes E6 and/or E7. Cellular RNA was extracted (Trizol, Invitrogen, Carlsbad, CA, USA) and submitted to reverse transcription (GoScript™ Reverse Transcription System, Promega Corporation, Madison, USA). cDNA samples were analysed by real-time PCR (GoTaq® qPCR Master Mix System, Promega Corporation, Madison, USA) using 100nM of each primer (GRPR *forward:* gcaccaaccagaccttcatt and *reverse:* tccacgggaagattgtaagc; GAPDH *forward:* caagatcatcagcaatgcctcc and *reverse:* gactgtggtcatgagtcctccc). Reactions were performed using ABI 7500 equipment (Applied Biosystems, USA).

All hematoxylin-eosin paraffin-embedded sections were reviewed by two independent pathologists and the best representative samples were identified. Biopsy samples, including punch biopsies, large loop procedures, conizations, or hysterectomies, were collected for the study. Fixed and paraffin embedded tissues were first cut in 3 μm sections and hematoxylin-eosin stained for selection of the appropriated tissue area. This study included 208 cervical samples, including 59 cases of cervicitis, 28 CIN3, 44 SCC, and 77 ADC.

All cervical samples were analyzed for the presence of HPV DNA. For DNA extraction, several 5 μm sections of the paraffin-embedded samples were collected in 1.5 ml microtubes. Samples were treated with xylene and digested with proteinase-K according to standard protocols described previously.^(23)^ The microtome blade was changed after each block was cut and all the surrounding area and apparatus were cleaned with xylene and ethanol after each sample processing to avoid contamination between the samples. DNA quality was checked by amplification of the human β-globin gene using PCO3+/PCO4+ primers.^(24)^ HPV detection and genotyping were performed using generic primers (GP5+/GP6+) and type specific probes (6, 11, 16, 18, 31, 33, 35, 39, 42, 45, 51, 52, 53, 54, 55, 56 and 58), respectively, as previously described.^(25)^

Immunohistochemical assays were performed by automated reactions using the UltraView Universal DAB Detection Kit^®^ (Ventana Medical Systems, Inc., Roche, Tucson, AZ, USA) and the Ventana BenchMark GX equipment (Roche Diagnostics, Mannheim, Germany), according to the manufacturer's instructions. In summary, the slides were submitted to an antigenic recovery process, carried out with a tris-based buffer, pH 8.0 (Ultra Cell Conditioning Solution 1, Roche Diagnostics, Mannheim, Germany) under heat, for 30 min. Next, to detect GRPR, rabbit polyclonal antibodies (Ziel A#01, clone ESTNQTFISCAPYPHSN, Ziel Biosciences, Brazil) were used at a 1:300 concentration and incubated for 32 min. Positive reactions were visualized with a cocktail (UltraView Universal HRP Multimer, Roche Diagnostics, Mannheim, Germany) containing peroxidase-conjugated anti-mouse and anti-rabbit secondary antibodies in the presence of DAB, resulting in a brown precipitate. The slides were counterstained with hematoxylin for 20 min. This entire process was performed inside the Ventana BenchMark GX equipment. As a positive control, a tissue known to express GRPR protein was incubated with the anti-GRPR antibody (as described above). The negative reaction control was obtained by incubating the same tissue with the antibody diluent alone. Immunohistochemical assays were evaluated considering the intensity and diffusion of stained tumor cells. A score of 1 to 3 was used for both GRPR staining intensity and extension, and these values were multiplied to generate a GRPR score. GRPR was categorized based on overall cellular positive reactions and classified as weak (extension and intensity score product <4) and strong (extension and intensity score product >4). This cut-off was determined according to ROC (Receiver Operating Characteristic) curve. Immunohistochemical evaluation was performed independently and blindly by two observers (AD- LO); discordant findings were discussed among them to achieve a consensus score.

All statistical analyses were performed with the Statistical Package for Social Sciences (SPSS) for Windows version 25.0. For experiments involving cell lines, the values obtained from biological replicates were evaluated through non-parametric tests (Kruskal Wallis). About human tissues, samples diagnosed as non-neoplastic were used as the reference category for comparisons with CIN3, SCC, and ADC. Furthermore, samples diagnosed as CIN3 and SCC were used as reference for ADC evaluation. Fisher's exact test was used to evaluate the association between GRPR expression, HPV infection and cervical lesion severity. The statistical tests were considered significant at p < 0.05.

This study was conducted in accordance with The Code of Ethics of the World Medical Association (Declaration of Helsinki), printed in the British Medical Journal (18 July 1964). Tissue samples were originally collected for diagnostic purposes and were anonymized before use. University of Campinas institutional ethics committee approved this research (04500146000-10) and waived the need for signed consent because this was a risk-free retrospective study, and it was no longer possible to contact many of the enrolled women.

## Results

GRPR mRNA levels were not significantly increased in HPV-positive cervical cancer-derived cell lines compared to normal keratinocytes. However, GRPR protein levels were higher in organotypic cultures derived from keratinocytes transduced with HPV16 oncogenes compared to those established from normal keratinocytes. Importantly, the percentage of samples expressing high GRPR levels increased progressively according to cervical lesion severity. Detailed discussion on the principal findings is depicted in the next sections.

### GRPR mRNA expression is not increased in cells expressing HPV16 or HPV18 oncogenes

**Figure 1:**
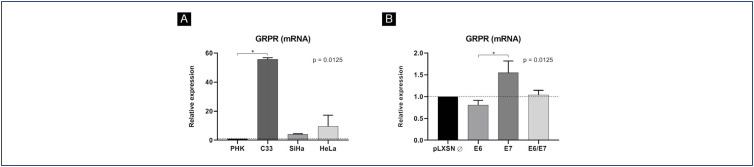
Relative expression of GRPR mRNA by real time PCR: Relative expression of GRPR mRNA in (A) normal keratinocytes (PHK) and cervical cancer-derived cell lines (C33, SiHa and HeLa) and (B) keratinocytes transduced with the empty vector (pLXSN Ø) or with the vector containing HPV16 oncogenes E6 and/or E7

GRPR mRNA levels in normal keratinocytes, keratinocytes transduced with HPV16 oncogenes E6 and/or E7, and cervical cancer-derived cell lines (C33, SiHa and HeLa) were analyzed using real-time PCR. All experiments were performed in biological triplicates. A greater GRPR mRNA expression was observed in cervical cancer derived cell lines compared to normal cells, although this difference was only evidenced in the HPV-negative C33 cell line (Figure 1A). The same was not seen between keratinocytes expressing HPV16 oncogenes and control cells containing the empty vector. Among keratinocytes, those expressing the E7 oncogene alone showed higher GRPR mRNA levels compared to cells expressing E6, but this was not significantly different from the control cells (Figure 1B).

### GRPR protein expression is upregulated in organotypic cell cultures expressing HPV16 oncoproteins

**Figure 2:**
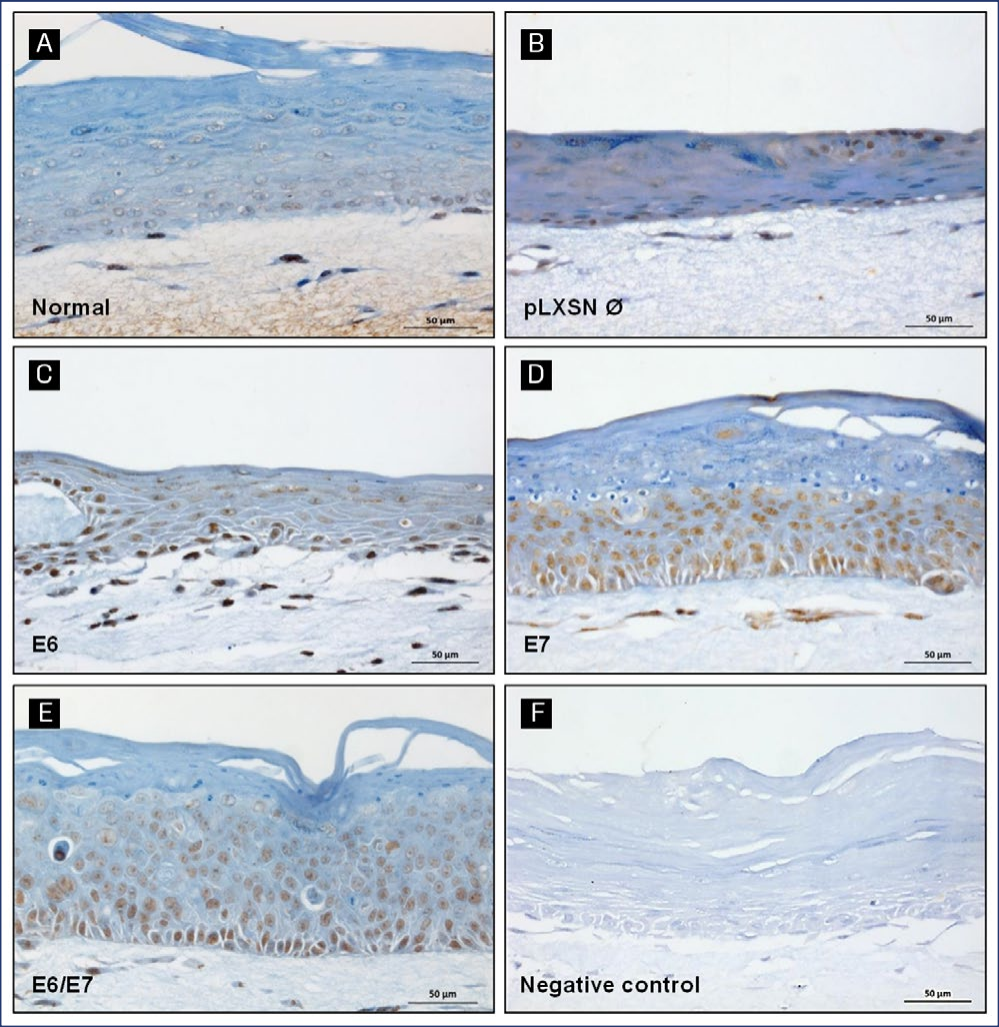
GRPR protein expression in organotypic cell cultures. GRPR protein expression was evaluated by immunohistochemistry in organotypic cell cultures sections derived from (A) normal keratinocytes; (B) keratinocytes transduced with the empty vector pLXSN (pLXSN Ø); (C) keratinocytes transduced with HPV16 E6 oncogene; (D) keratinocytes transduced with HPV16 E7 oncogene; (E) keratinocytes transduced with HPV16 E6 and E7 oncogenes. (F) Negative control: normal keratinocytes without primary antibody

We also explored the relation between GRPR and the expression of HPV oncogenes. The idea was to understand if this protein levels were, in some way, influenced by E6 and/or E7. To achieve this goal, we used a specific anti-GRPR antibody developed by Ziel Biosciences (São Paulo, Brazil). In organotypic cell cultures derived from normal keratinocytes or from keratinocytes transduced with pLXSN empty vector, GRPR protein expression was weak and localized mainly to the basal and parabasal epithelial layers (Figures 2A and 2B). On the other hand, cultures derived from keratinocytes expressing HPV16 oncogenes, particularly E7, showed a strong and predominantly nuclear GRPR staining, distributed throughout the entire epithelial layers. In these cultures, HPV oncogene expression altered tissue architecture and normal differentiation, as seen in previous reports (Figures 2C and 2D). ^(26,27)^

### Immunohistochemical evaluation of GRPR protein in cervical samples

**Figure 3:**
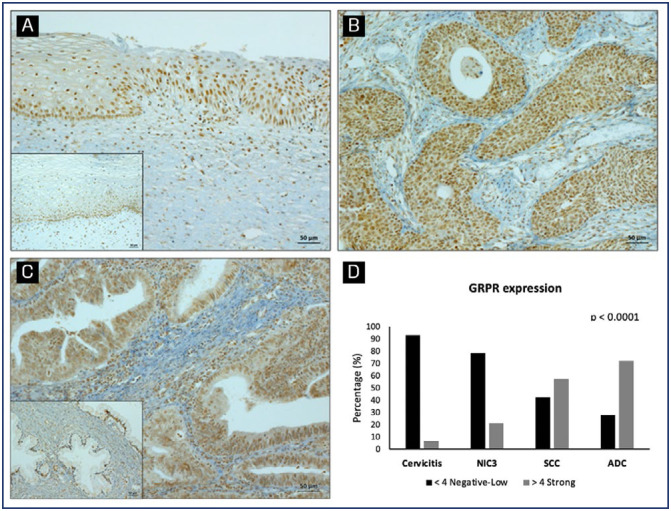
GRPR protein expression in cervical samples. Representative GRPR protein expression determined by immunohistochemistry in (A) transition area to a high-grade lesion; (B) squamous cell carcinoma and (C) adenocarcinoma. Images in-set: cervicitis (A) and normal glandular tissue (C). Graphic representation of GRPR expression in cervical tissue samples according to the histopathological diagnosis. Samples were analyzed according to GRPR staining intensity and grouped into negative-low or strong groups. The percentage of samples allocated in each group according to histological classification is shown (D). *p < 0.0001 indicates a significantly different distribution between the analyzed variables (GRPR, cervicitis, NIC3, SCC and ADC) (Fisher's exact test)

GRPR protein levels and expression pattern was determined in 208 cervical samples by immunohistochemistry. A representative staining of GRPR in different samples is shown in figures 3 A, B and C. GRPR expression was graded as scores into weak (≤ 4) or strong (>4). Strong GRPR expression increased with cervical lesion severity (p<0.001), with 6.7% of cervicitis, 21.4% of CIN3, 57.5% of SCC, and 72% of ADC samples showing high GRPR levels. Conversely, weak GRPR expression occurred in 93.3%, 78.6%, 42.5%, and 28% of cervicitis, CIN3, SCC, and ADC, respectively (Figure 3D).

### HPV prevalence and GRPR expression in cervical samples

**Figure 4:**
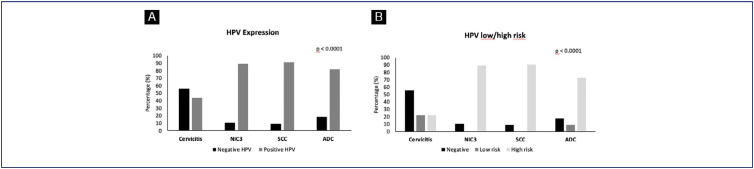
HPV DNA prevalence in cervical tissue samples according to the histopathological diagnosis. Samples were analyzed according to HPV detection and grouped into negative or positive HPV groups (A). The classification into low and high-risk HPV was also performed (B). The percentage of samples allocated in each group, according to histological classification, is shown. *p < 0.0001 indicates a significantly different distribution between the analyzed variables (HPV, cervicitis, NIC3, SCC and ADC) (Fisher's exact test)

**Figure 5:**
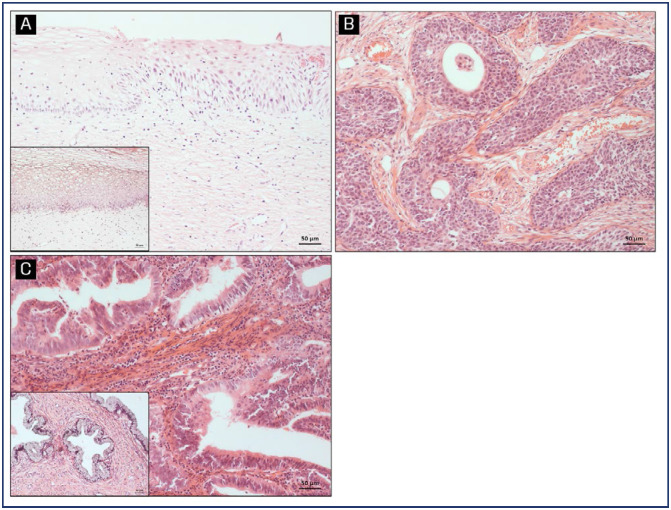
Correspondent Hematoxylin and Eosin (H&E) images of pictures included in figure 3. (A) transition area to a high-grade lesion; (B) squamous cell carcinoma and (C) adenocarcinoma. Images in-set: cervicitis (A) and normal glandular tissue (C)

The prevalence of HPV infection and type distribution (low and high-risk) in relation to histopathological diagnosis are shown in figure 4. Approximately 44% of women with non-neoplastic diagnoses tested positive for HPV (Figure 4A), equally distributed between high and low risk types (Figure 4B). HPV was detected in 89.3% of CIN3 and 90.9% of SCC samples, all of them classified as high-risk HPV. In ADC samples, 81.8% tested positive for the virus, with only 9.1% being low-risk HPV (Figures 4 A and B). Notably, GRPR staining was predominantly observed in cell nuclei of CIN3 lesions, while normal epithelium showed positivity in the basal layer. In samples of invasive carcinomas and adenocarcinomas, both nuclear and cytoplasmic GRPR staining was observed. These GRPR staining differences in the cell compartments brought up the especulation if it could be associated to the invasion process (Figure 5).

Table 1 shows the analysis of HPV (low and high-risk) detection relative to GRPR score. There was not a statistically significant association between these two variables in cervical samples.

**Table 1:** GRPR expression (score) *versus* HPV detection in cervical samples

		GRPR expression		Total* n	p-value
		<4 Negative/Weak n(%)	>4 Strong n(%)		
HPV	Negative	20(22.7)	13(15.3)	33	0.248
	Positive	68(77.3)	72(84.7)	140	
	Total*	88	85	173	

* HPV DNA results were not available for 35 patients

## Discussion

Our results evidenced widespread GRPR expression in cervical cancer and low expression in non-malignant samples. GRPR expression is highly predictive of cervical lesion severity, regardless of HPV infection. These findings suggest that GRPR score might be used in combination with cytology and HPV testing as a valuable tool for characterizing cervical precursor lesions and cervical cancer. In a similar way, GRPR has been shown to be overexpressed in several tumors.^(28-30)^ High expression of GRPR protein was evidenced in breast cancer, with tumors positive for hormonal receptors expressing higher GRPR mRNA levels than normal tissues.^(28)^ In anal canal carcinomas, for example, GRPR presented a diffuse and homogenous pattern of expression compared to control samples, suggesting a possible role of this receptor in carcinogenesis.^(29)^

Previous studies reported a strong GRPR staining in cervical cancer samples by immunohistochemical analysis and raised the hypothesis that GRPR expression could serve as a valuable diagnostic marker for early detection of this type of tumor.^(15,21)^ However, there is limited available data exploring GRPR expression in cervical neoplasia and none of the previous studies evaluated the correlation between HPV infection and GRPR in this scenario.

We first analyzed GRPR mRNA levels in cervical cancer-derived cell lines and in keratinocytes expressing HPV16 E6 and/or E7. Interestingly, C33 cells, an HPV-negative cervical cancer-derived cell line that harbor mutations in p53 and pRb genes, exhibited high levels of GRPR mRNA compared to normal keratinocytes. On the other hand, no significant differences in GRPR mRNA levels were observed between HPV-transformed cells, keratinocytes expressing HPV16 oncogenes and normal keratinocytes. Although E7 was associated with higher levels of GRPR mRNA than E6, no difference was found compared to control cells. These observations suggest that HPV is not mandatory to explain the increasing of GRPR at the mRNA level and that post transcriptional events may be involved in the regulation of GRPR protein expression.

Subsequently, to determine the effects of cell differentiation and HPV oncogenes on the regulation of GRPR protein levels, we analyzed its expression in organotypic cell cultures established from control keratinocytes and keratinocytes transduced with E6 and/or E7. We showed that GRPR was upregulated in cultures containing HPV16 oncogenes. Besides, our results suggest that this effect is mainly associated with E7 expression.

When analyzing GRPR protein expression in cervical samples, we observed a strong association between its levels and cervical disease severity. This findings aligns with earlier studies that linked GRPR to cervical dysplasia and invasive squamous cell carcinoma of the cervix.^(15,21)^ Recently, Kunos et al. (2023)^(31)^ showed that GRPR was overexpressed in 100% of adenocarcinoma and 63% of primary squamous cell carcinoma, as determined by staining intensity immunoreactive score (IRS). Besides, it was observed that GRPR ligands have the ability to both stimulate and inhibit cell line proliferation in breast, ovarian and cervical cancer cell lines.^(14,22,31)^ Taking together, these findings provide evidence suggesting a potential role of GRPR in cervical cancer development.

Currently, Pap test is the standard method for detecting cervical cancer. However, its low sensitivity and the interobserver discrepancies in morphological interpretation remain relevant and very disturbing points of concern regarding its use as a screening method.^(32,33)^ Thus, there is a need for a more effective diagnostic tool that improves Pap test interpretation.

As expected, our results confirmed that the detection rate of high-risk HPV correlates directly with cervical disease, consistent with previous studies.^(14,34)^ HPV DNA testing has been introduced alone or together with other tests as an alternative for screening.^(11-13)^ However, most HPV infections are harmless, and the presence of HPV does not directly identify cervical precancer lesions, but indicates an increased risk of cervical cancer. Additional tests to detect precancerous lesions and/or cancer are needed to enhance screening specificity.^(14)^

HPV infection is recognized as the primary etiological factor contributing to the development of cervical cancer.^(14)^ As anticipated, in our cohort, HPV DNA positivity was not associated with GRPR in cervicitis, CIN3, SCC, and ADC samples. Nonetheless, emerging evidence suggest that GRPR may play a role in cervical carcinogenesis, as it has been shown to modulate cell viability by exerting both inhibitory and stimulatory effects.^(15,21)^ Further investigations are warranted to elucidate the specific signaling pathways implicated in cervical cancer tumorigenesis, shedding light on the interplay between HPV infection and GRPR-mediated processes. To the best of our knowledge, this is the first study addressing the relation between HPV infection and GRPR in cervical samples. Although no association was found, an increased expression of GRPR was observed with disease progression.

The immunohistochemistry procedure is a feasible and affordable method to be used in cancer research and clinical diagnosis, and herein demonstrated potential usefulness to recognize the role of GRPR as a promising marker for early identification, management and monitoring of cervical intraepithelial neoplasia.^(15,21)^ It is important to highlight that, in the present study, a specific anti-GRPR antibody developed by Ziel Biosciences (Patent number BR 10 2014 007315-9) was used for immunohistochemical analysis, and the results obtained through the categorization of GRPR expression scores presented relevant results regarding sensibility and specificity. Therefore, the use of anti-GRPR specific antibody and GRPR score could enhance the detection of precursor lesions and cancer and improve the diagnosis, patient management, and monitoring of disease progression.

## Conclusion

Our results indicate that GRPR expression is highly predictive of cervical lesion severity, irrespective of HPV infection, and might contribute to improve diagnosis and therapeutic management as well as serve as a marker of disease progression.
